# Examining alcohol use disorder recovery definitions and demographic reporting in studies of structural brain recovery: an updated systematic review and extension

**DOI:** 10.1093/alcalc/agag044

**Published:** 2026-07-21

**Authors:** Catherine D Trinh, Alana Egan, Rachel Girard, Sydney Iacoi, Diana Ho, Emily K Carter, Nathan E Cook, Nichea S Spillane

**Affiliations:** Department of Psychology, University of Rhode Island, 142 Flagg Rd, Chafee Hall, Kingston, RI 02881, United States; Department of Psychology, University of Rhode Island, 142 Flagg Rd, Chafee Hall, Kingston, RI 02881, United States; Department of Psychology, University of Rhode Island, 142 Flagg Rd, Chafee Hall, Kingston, RI 02881, United States; Department of Psychology, University of Rhode Island, 142 Flagg Rd, Chafee Hall, Kingston, RI 02881, United States; Department of Psychology, University of Rhode Island, 142 Flagg Rd, Chafee Hall, Kingston, RI 02881, United States; Department of Psychology, University of Rhode Island, 142 Flagg Rd, Chafee Hall, Kingston, RI 02881, United States; Department of Psychology, University of Rhode Island, 142 Flagg Rd, Chafee Hall, Kingston, RI 02881, United States; Department of Psychology, Arizona State University, 950 S. McAllister Ave, Psychology Building, Tempe, AZ 85281, United States

**Keywords:** neuroimaging, alcohol use disorder, recovery, prefrontal cortex, review

## Abstract

**Introduction:**

The National Institute on Alcohol Abuse and Alcoholism’s (NIAAA) definition of recovery from alcohol use disorder (AUD) expanded from an abstinence-based model to encompass remission from AUD symptoms and cessation from heavy drinking. However, many neuroimaging studies continue to use abstinence-based definitions of recovery. Many neuroimaging studies also report limited sample demographic information and diverse participants remain underrepresented in neuroimaging research. The aims of this systematic review were to: (i) update a review by Parvaz et al. (2022) on the topic of structural changes in the prefrontal cortex, (ii) apply the NIAAA’s updated definition of recovery to studies in the original review and newly identified studies, and (iii) report demographic information from all included studies.

**Materials and Methods:**

The original review was extended by searching PubMed and PsycInfo for articles published between May 2021 and June 2025. Demographic reporting practices were reviewed and the Newcastle-Ottawa Quality Assessment Scale was used to assess bias in all included articles.

**Results:**

There was one newly identified study which reported cessation from heavy drinking. However, the 19 original studies were abstinence-based and did not use the updated NIAAA definition of recovery. Demographic information in the 20 included studies was largely limited to age, sex assigned at birth, and educational attainment. Reporting on additional demographic information was scarce.

**Conclusions:**

Future neuroimaging studies should examine duration of alcohol use disorder symptom remission and cessation of heavy drinking as outcomes. Moreover, studies should include additional demographic information. These efforts would align with national guidelines and advance clinical science.

## Teaser

**Short Summary:** Only one newly identified study reported duration of heavy drinking cessation. None of the included studies reported duration of remission from AUD symptoms. While most studies included age, sex, and education information, reporting on racial identity, Hispanic or Latine identity, socioeconomic status, and differentiation between sex and gender was limited.

## Introduction

Alcohol use has been identified as a major risk factor for morbidity and mortality. These health risks increase in a dose response manner (Griswold et al. 2018, Daviet et al. 2022). In 2023, alcohol use disorder (AUD) affected 28.9 million people (or 10.2% of the population) in the United States who were ages 12 years or older (SAMHSA 2024). In individuals diagnosed with an AUD, greater impulsivity is associated with higher relapse rates and treatment dropout (Loree et al. 2015). Neuroimaging studies have consistently identified a robust association between AUD and structural differences in specific brain regions, especially in the prefrontal cortex which is involved in controlling impulsive behaviors (Yang et al. 2016, Wang et al. 2016b, Durazzo and Meyerhoff 2020). Long-term alcohol use is associated with increased inflammatory signaling, which, in turn, is associated with structural damage (Lanquetin et al. 2021, Adams et al. 2023) and decreased cortical thickness (Karoly et al. 2021). A meta-analysis comparing people with AUD to controls found that those with AUD showed decreased gray matter volume in multiple regions, including the prefrontal cortex, and that duration of AUD was associated with having less gray matter volume in these regions (Yang et al. 2016). Even among generally healthy individuals without AUD, increased alcohol use was negatively associated with global gray matter volume, particularly in frontal regions such as the prefrontal cortex, as well as white matter microstructure (Daviet et al. 2022). Although many of these neuroimaging studies are cross-sectional, these findings suggest associations between alcohol use, frontal lobe dysfunction, and impulsivity among people with AUD (Wang et al. 2016b). Recovering from AUD may involve structural changes to gray and white matter in the prefrontal cortex; such frontal lobe recovery may be especially promising as this might be associated with decreased relapse rates and improved treatment outcomes for people with AUD (Loree et al. 2015).

Most individuals with AUD experience improved functioning and well-being (Tucker et al. 2020) or decrease their AUD-related consequences over time through natural recovery without the assistance of treatment (Witkiewitz and Tucker 2020). The National Institute on Alcohol Abuse and Alcoholism (NIAAA) defines recovery as both a process and an outcome: “Recovery is a process through which an individual pursues both remission from alcohol use disorder (AUD) and cessation from heavy drinking. Recovery can also be considered an outcome such that an individual may be considered ‘recovered’ if both remission from AUD and cessation from heavy drinking are achieved and maintained over time” (Hagman et al. 2022, p. 808; NIAAA n.d.). The NIAAA specifies that an individual is in remission if they were previously diagnosed with AUD but no longer meet any criteria for AUD symptoms [as defined by the Diagnostic and Statistical Manual of Mental Disorders (DSM-5)] besides craving alcohol (NIAAA n.d.). Durations for remission from AUD include: “initial” (for at least 3 months in the same year), “early” (3 months to 1 year), “sustained” (1–5 years), and “stable” remission (>5 years; Hagman et al. 2022; NIAAA n.d.; Tyburski et al. 2014). The NIAAA definition of cessation from heavy drinking stipulates consuming no >14 drinks per week (or four drinks per day) for men and no >7 drinks per week (or three drinks per day) for women (NIAAA n.d., Hagman et al. 2022). Similar to the duration of remission based on DSM-5 AUD criteria, duration of cessation from heavy drinking is also divided into various categories including: “initial” (at least 3 months in the same year), “early” (3 months to 1 year), “sustained” (1–5 years), and “stable” (>5 years) periods (NIAAA n.d.). Taken together, the NIAAA defines recovery as including both remission from AUD symptoms and cessation of heavy drinking, which represents a departure from previous definitions of recovery.

The recent broadening of the definition of recovery reflects a harm reduction approach as it recognizes that recovery exists beyond abstinence-based outcomes, such as in controlled drinking strategies that have emerged as a viable treatment option (Henssler et al. 2021). Abstinence-based recovery has been associated with improved quality of life, longer length of time in recovery (Subbaraman and Witbrodt 2014), as well as greater functioning and well-being (Eddie et al. 2022). Yet, because the field of alcohol research has long focused on populations that have abstinence-based recovery goals, most research with people who are recovering from AUD rely on a definition of recovery that requires complete abstinence from alcohol or the absence of AUD symptoms for inclusion (Witkiewitz et al. 2020, Witkiewitz and Tucker 2020). Alcohol consumption and AUD symptoms occur on a continuum of severity rather than a binary “abstinent versus not-abstinent” or “has AUD symptoms versus does not have AUD symptoms” distinction (Witkiewitz and Tucker 2020). The inclusion of participants who return to drinking alcohol in neuroimaging studies, as well as the measurement of alcohol consumption and AUD symptoms, would more comprehensively capture the range of alcohol use and AUD symptom severity during recovery.

In addition to the use of restrictive abstinence-based inclusion criteria outcomes, another limitation of prior neuroimaging studies is the under-representation of people with diverse sexual, gender, racial, and ethnic identities, as well as socioeconomic statuses. The over-representation of White, non-Hispanic or Latino (Ricard et al. 2023), cisgender, and heterosexual (Edmiston and Juster 2022) participants in neuroimaging research may exacerbate inequities in effective healthcare procedures and treatments, as these practices are informed by emerging research that is non-representative (Ricard et al. 2023). Although individuals from all demographic groups in the United States use alcohol, studies have found significant disparities in frequency of alcohol use, quantity of alcohol use, negative alcohol-related health consequences, and diagnoses of AUD across subgroups (Delker et al. 2016, White 2020). For example, while White participants report the most alcohol consumption in national surveys, AUD and negative alcohol-related health consequences are more prevalent among Native American and Black American participants (Delker et al. 2016). Moreover, though males have historically consumed more alcohol at higher frequencies than females, females have reported larger increases in negative alcohol-related health consequences in recent years (White 2020). Thus, reporting demographic information in studies is a step toward examining potential health disparities and developing research practices that are representative (Bibbins-Domingo et al. 2022, Müller et al. 2023, Ricard et al. 2023). Furthermore, individuals who hold minoritized identities may undergo chronic stressors such as experiencing bias, stigma, racism, and discrimination, which can negatively impact brain health and affect neuroimaging findings (Grasser and Jovanovic 2022). To illuminate and address these disparities, it is not only best practice to collect and report comprehensive participant demographic data, but also vital to investigate how the experiences of individuals with minoritized identities might relate to alcohol use in neuroimaging research.

A recent review by Parvaz et al. (2022) on the relationship between alcohol, other substance use, and structural and functional recovery reported a positive association between alcohol abstinence and structural brain recovery in multiple regions, including the frontal, parietal, temporal, and occipital lobes, and the thalamus, insula, hippocampus, cerebellum. Of note was the finding that increased gray matter and cortical thickness in the frontal regions was associated with alcohol abstinence. Further, the longer the duration of time between brain scans (which was used as a proxy for length of abstinence and/or decreased alcohol use), the greater the improvements in these brain areas (Parvaz et al. 2022). While these promising findings suggest that early abstinence might be associated with recovery in multiple regions, including frontal regions, the review had several limitations. First, the review did not examine demographic reporting practices of the included studies. Second, although the review aimed to study recovery from AUD, it did not utilize NIAAA’s expanded definitions of AUD recovery that specify duration of time in remission and cessation of heavy drinking (NIAAA n.d.). These are important gaps that warrant attention as they reflect current best practices in the field. Moreover, the Parvaz et al. (2022) review included studies published up to May 2021. Therefore, the goals of the current systematic review are to: (i) update the original literature search conducted by Parvaz et al. (2022), (ii) apply the NIAAA definition of recovery to characterize structural brain recovery based on duration of remission and cessation from heavy drinking in both newly found and original studies that were previously included in the Parvaz et al. (2022) review, and (iii) report the demographic information of participants in the included studies.

## Materials and methods

### Search strategy

**Figure 1:**
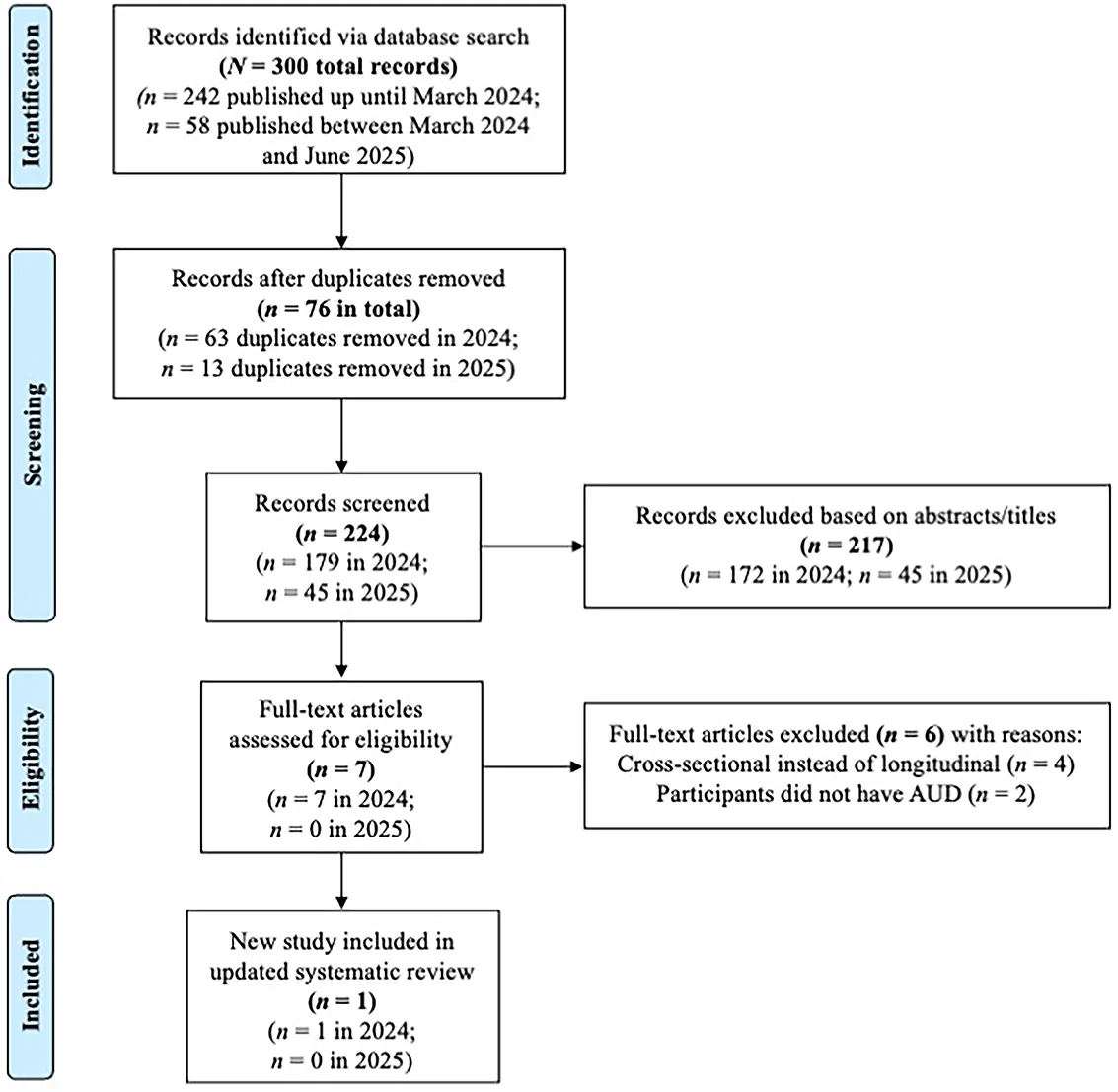
The PRISMA flow diagram shows the number of records identified (*N* = 300), screened (*n* = 224), excluded (*n* = 223), and included in the final review (*n* = 1).

Following Preferred Reporting Items for Systematic Reviews and Meta-Analyses (PRISMA) guidelines, a systematic review was conducted of structural neuroimaging studies on the prefrontal cortex of adults in recovery from AUD published since May 2021, which was the date of the literature search in the original review by Parvaz et al. (2022). This systematic review and review protocol were pre-registered in PROSPERO under ID #CRD42024512223, entitled “Frontal brain recovery for adults with alcohol use disorder across periods of remission and heavy drinking cessation: An update and extension.” PubMed and PsycInfo were initially searched on 1 March 2024, and again on 1 June 2025 (with filters set to show results from 1 March 2024, onwards). The search strategy is depicted in the flow diagram (Fig. 1).

The following search terms were used to replicate the original review and were not MeSH terms. These terms were entered into PubMed and PsycInfo:

(“magnetic resonance imaging” OR “MRI” OR “diffusion tensor imaging” OR “DTI” OR “structural” OR “volume” OR “volumetric”) AND (“alcohol” OR “alcohol use disorder” OR “alcohol addiction” OR “alcohol dependence” OR “alcoholism” OR “alcohol problem” OR “alcohol consequence”) AND (“prefrontal cortex” OR “PFC”)

Following the methods outlined in Parvaz et al. (2022), articles were included if they fulfilled the following criteria:

1. Reporting in English language
2. Employed a structural neuroimaging method
3. Participants were adults who met criteria for AUD (by DSM-IV-TR, DSM-V, or ICD-10 criteria)
4. Second follow-up scan conducted at least 24 hours following a first scan
5. Second follow-up occurred after a period of abstinence/reduced drinking or fewer AUD symptoms
6. At least 10 participants at the follow-up scan
7. Original empirical article published in a peer-reviewed journal

Articles were excluded if:

1. A pharmacological or psychotherapeutic intervention was utilized (i.e. clinical trial)
2. They were a meta-analysis, review (e.g. narrative, scoping, systematic), case study, or unpublished thesis/dissertation

Two authors (C.D.T. and A.E.) screened the abstracts and titles that were searched on 1 March 2024, for inclusion, and a third author (R.G.) resolved discrepancies. Author consensus for this first round was 94%. For the abstracts and titles that were searched on 1 June 2025, two authors (R.G. and S.I.) screened for inclusion, and a third author (A.E.) resolved one discrepancy. Author consensus for this second round was 98%.

#### Data extraction

Following initial screening, one author (C.D.T.) examined the full text of the one newly identified article and the 19 articles from the original Parvaz et al. (2022) review to extract information such as participant demographic data (age, sex assigned at birth, race, ethnicity, educational attainment), and NIAAA recovery definitions (duration of remission and heavy drinking cessation). Of note, several articles used language to describe a recurrence of alcohol use that may be stigmatizing (Miller 2015, Hartwell et al. 2022) by labeling these individuals as “relapsers” or “alcoholics”. The authors of the current study made the decision to use present-day standards throughout this manuscript to describe individuals who had a recurrence of alcohol use or returned to alcohol use. We will note when we have replaced the terms “relapsers” or “alcoholics” from the original texts with an asterisk.

#### Quality assessment

Three authors (D.H., E.K.C., and N.E.C.) completed a risk of bias assessment for the included studies using the Newcastle-Ottawa Quality Assessment Scale (NOS) for cohort studies (Wells et al. n.d.). The NOS is an 8-item checklist that evaluates the quality of non-randomized studies (case control and cohort studies) based on the selection of the study groups (“selection”), comparability between groups (“comparability”), and outcome of interest (“outcome”, which is specific to cohort studies). The NOS is scored by awarding one point per checklist item fulfilled, with possible total points equaling 4 for selection, 2 for comparability, and 3 for outcomes (Gierisch et al. 2014), and a total summed score ranging from 0 to 9 for each article. Higher scores indicate stronger methodological quality/lower risk of bias. The NOS has demonstrated face validity, content validity, and inter-rater reliability (Wells et al. n.d.), though one study found varying inter-rater reliability and fair reliability for the overall score (Hartling et al. 2013).

## Results

### Search results

A total of 242 articles were located during the database search in March 2024. After 63 duplicates were removed, 179 unique articles remained. Following abstract and title review, 172 were excluded for not meeting inclusion criteria due to the following reasons: incompatible population (*n* = 110), study design (*n* = 40), publication type (*n* = 16), and study duration (*n* = 6). The remaining 7 articles were reviewed in full, and an additional 6 articles were excluded due to incompatible study design (being cross-sectional instead of longitudinal; *n* = 4) or population (participants did not have AUD; *n* = 2). Thus, only one new article by Maillard et al. (2022) was included from this search. A second database search conducted in June 2025 yielded 58 articles. After 13 duplicates were removed, 45 unique articles remained. However, all 45 articles were excluded following abstract and title review due to the following reasons: incompatible population (*n* = 29), study design (*n* = 15), and publication type (*n* = 1). No new articles were included from this second search. See Fig. 1 for the number of records identified, screened, excluded, and included in the final review based on PRISMA guidelines.

### Quality assessment scores

The quality of the one new study identified from the systematic literature search (Maillard et al. 2022) and the 19 studies from the original Parvaz et al. (2022) review were assessed using the NOS for cohort studies (Wells et al. n.d.). Overall, the 20 studies received a total average of 2.1 (out of 4) in selection, 1.4 (out of 2) in comparability, and 2.2 (out of 3) in outcome. Table 1 depicts the individual quality assessment scores that each study received in the three categories.

**Table 1:** Newcastle-Ottawa quality assessment scores.

Article	Selection (0–4)	Comparability (0–2)	Outcome (0–3)
Maillard et al. (2022)	2	2	2
Alhassoon et al. (2012)	2	2	3
Bach et al. (2020)	2	2	2
Cardenas et al. (2007)	2	0	2
Demirakca et al. (2011)	2	2	2
Durazzo et al. (2015)	2	1	2
Durazzo et al. (2017)	2	2	2
Durazzo and Meyerhoff (2020)	2	2	3
Gazdzinski et al. (2005)	2	1	2
Gazdzinski et al. (2008)	2	1	3
Gazdzinski et al. (2010)	2	1	2
Hoefer et al. (2014)	2	2	2
Kühn et al. (2014)	2	1	2
Mon et al. (2013)	2	1	2
Pfefferbaum et al. (1995)	2	1	2
Pfefferbaum et al. (2014)	3	1	3
Shear et al. (1994)	2	0	3
van Eijk et al. (2013)	2	2	1
Wang et al. (2016a)	2	2	2
Zou et al. (2018)	2	1	2
**Average scores (all articles)**	2.1	1.4	2.2

*Note*. Values indicate the “quality” of each article within the following categories: “selection” of study groups (possible scores range from 0-4), “comparability” between groups (possible scores range from 0-2), and the “outcome” of interest (possible scores range from 0-3).

### Summary of demographic information

Table 2 summarizes demographic information from the newly included study by Maillard et al. (2022), as well as the 19 original studies from the original Parvaz et al. (2022) review. Overall, most studies reported information on age, sex, and education. No studies differentiated between sex assigned at birth and gender. Only 10 studies provided any information on racial characteristics, and even fewer (*n* = 3) provided information on Hispanic or Latine participants. One study (Pfefferbaum et al. 2014) reported information on socioeconomic status between participants with AUD and controls; the rest did not report socioeconomic status.

**Table 2:** Summary of demographic information from included studies.

Article	*n* (baseline)	Age in years, *M* (*SD*)	Sex assigned at birth (%)	Race (%)	Ethnicity (%)	Education in years, *M* (*SD*)
Maillard et al. (2022)	AUD: 54 CON: 36	AUD: 46 [39–54]^**^ CON: 45 [39–47.25]^**^	Female: 17% Male: 83%	NR	NR	AUD: 11 [10–13.5]^**^ CON: 12 [11–12]^**^
Alhassoon et al. (2012)	AUD: 15 CON: 15	AUD: 51.4 (6.0) CON: 51.8 (7.4)	Female: 0% Male: 100%	AUD: 13% non-White CON: 6% non-White	NR	AUD: 13.6 (2.4) CON: 13.9 (2.6)
Bach et al. (2020)	AUD: 62 CON: 74	AUD: 47.6 (9.7) CON: 44.12 (9.6)	Female: 22% Male: 78%	NR	NR	NR
Cardenas et al. (2007)	AUD: 47 LD: 18	AUD: 45 (8) LD: 49 (14)	Female: 8% Male: 92%	AUD: 71% White, 13% African–American LD: “similar” to AUD	NR	AUD: 14 (2) LD: 17 (2)
Durazzo et al. (2015)	AUD: 50 CON: 66	AUD: 46.6 (8.2) CON: 45.0 (10.1)	Female: 47% Male: 53%	NR	NR	NR
Durazzo et al. (2015)	AUD + smoke: 46 AUD + no smoke: 36 CON: 32	AUD + smoke: 49 (9) AUD + no smoke: 52 (11) CON: 47 (9)	Female (overall): 10% AUD + smoke: 93% male AUD + no smoke: 88% male CON + no smoke: 91%	AUD + smoke: 74% White AUD + no smoke: 75% White CON + no smoke: 72% White	NR	AUD + smoke: 14 (2) AUD + no smoke: 14 (2) CON + no smoke: 17 (3)
Durazzo et al. (2017)	Abstained: 42 Recurrence^*^: 72 CON: 33	Abstained: 52.0 (12.2) Recurrence^*^: 50.1 (8.2) CON: 46.1 (9.4)	Male: “predominately. . .male” AUD: 7% female CON: 6% female	Abstained: 80% White Recurrence^*^: 71% White CON: 73% White	NR	Abstained: 14.6 (2.2) Recurrence^*^: 13.4 (1.8) CON: 16.7 (2.5)
Durazzo and Meyerhoff (2020)	Abstained: 24 Recurrence^*^: 54 CON: 33	Abstained: 53 (9) Recurrence^*^: 51 (8) CON: 46 (9)	Abstained: 90% male Recurrence^*^: 92% male CON: 94% male	Abstained: 79% White Recurrence^*^: 74% White CON: 73% White	NR	Abstained: 14.6 (2.2) Recurrence^*^: 13.5 (1.9) CON: 16.7 (2.6)
Gazdzinski et al. (2005)	AUD: 18 CON: 17	AUD (baseline): 50.6 (9.3) CON (baseline): 45.0 (6.8)	AUD: 4% female, 96% male CON: 12% female, 88% male	NR	NR	AUD (baseline): 14.2 (2.2) CON (baseline): 16.2 (2.2)
Gazdzinski et al. (2008)	AUD + smoke: 13 AUD + no smoke: 11 LD + no smoke: 14	AUD + smoke: 50.7 (9.0) AUD + no smoke: 50.2 (9.1) LD + no smoke: 47.3 (8.2)	Female (overall): 0% AUD: 100% male LD + no smoke: 100% male	AUD + smoke: 69% White, 23% African American, 8% Native American, 0% Asian, 0% Pacific Islander/Polynesian AUD + no smoke: 73% White, 0% African American, 9% Native American, 0% Asian, 0% Pacific Islander/ Polynesian LD + no smoke: 71% White, 7% African American, 0% Native American, 14% Asian, 7% Pacific Islander/Polynesian	AUD + smoke: 0% Latino AUD + no smoke: 18% Latino LD + no smoke: 0% Latino	AUD + smoke: 13.9 (1.3) AUD + no smoke: 14.0 (2.6) LD + no smoke: 16.4 (2.6)
Gazdzinski et al. (2010)	AUD + smoke: 20 AUD + no smoke: 16 LD + no smoke: 22	AUD + smoke: 47.7 (9.5) AUD + no smoke: 51.5 (10.3) LD + no smoke: 48.3 (8.4)	Male: “mostly male” AUD + smoke: 5% female AUD + no smoke: 13% female LD + no smoke: 9%	NR	NR	AUD + smoke: 13.8 (2.0) AUD + no smoke: 14.3 (2.2) LD + no smoke: 17.1 (2.7)
Hoefer et al. (2014)	AUD + smoke: 49 AUD + no smoke: 35 LD + no smoke: 35	AUD + smoke: 49.6 (9.0) AUD + no smoke: 53.6 (10.5) LD + no smoke: 45.6 (9.9)	Male: “predominantly male” AUD + smoke: 3% female AUD + no smoke: 16% female LD + no smoke: 9%	AUD + smoke: 69% White, 20% African American, 3% other AUD + no smoke: 82% White, 4% African American, 3% other LD + no smoke: 71% White, 9% African American, 6% other	AUD + smoke: 7% Latino AUD + no smoke: 11% Latino LD + no smoke: 14% Latino	AUD + smoke: 13.5 (1.8) AUD + no smoke: 14.6 (2.4) LD + no smoke: 16.7 (2.4)
Kühn et al. (2014)	AUD: 42 CON: 32	AUD: 42.1 (11.6) CON: 40.8 (13.4)	AUD: 19% female, 81% male CON: 41% female, 59% male	NR	NR	NR
Mon et al. (2013)	AUD: 62 LD + no smoke: 17	50.8 (10.6) (AUD subsample)	Female: 15% Male: 85% (AUD subsample)	White: 84% African American: 11% Native American: <2% Asian: <2% Pacific Islander: <2% (AUD subsample)	Hispanic: 5% (AUD subsample)	14.3 (2.4) (AUD subsample)
Pfefferbaum et al. (1995)	AUD: 58 CON: 58	AUD: 45.0 (10.9) CON: 45.3 (14.2)	Female: 0% Male: 100%	NR	NR	AUD: 13.2 (2.6) CON: 16.3 (3.0)
Pfefferbaum et al. (2014)	AUD: 47 CON: 56	AUD: 44.3 (9.2) CON: 43.0 (10.1)	AUD: 40% female, 60% male CON: 57% female, 43% male	AUD: 62% White, 36% African American, 2% other CON: 64% White, 18% African American, 18% other	NR	AUD: 13.4 (2.3) CON: 15.2 (2.3)
Shear et al. (1994)	AUD (baseline): 24 Abstained (time 2): 15 Recurrence^*^ (time 2): 9	Abstained: 48.8 (9.6) Recurrence^*^: 48.6 (10.9)	Female: 0% Male: 100%	NR	NR	Abstained: 14.4 (1.9) Recurrence^*^: 13.9 (1.7)
van Eijk et al. (2013)	AUD: 49 CON: 55	AUD: 47 (10.1) CON: 45.3 (11.9)	AUD: 18% female, 82% male CON: 24% female, 76% male	NR	NR	NR
Wang et al. (2016a)	AUD: 49 CON: 20	AUD: 47.02 (10.00) CON: 46.65 (12.37)	AUD: 18% female, 82% male CON: 15% female, 85% male	NR	NR	NR
Zou et al. (2018)	AUD: 85 CON: 17	AUD: 50.5 (9.8) CON: 44.3 (9.9)	Female (overall): 20% AUD: 89% male CON: 88% male	AUD (timepoints 1–3): 77%–81% White CON (baseline): 71% White Non-White: 20%	NR	AUD: 13.9 (2.2) CON: 16.7 (2.4)

*Note*. NR = not reported; AUD = people with alcohol use disorder; CON = controls (people without alcohol use disorder); LD = people who drink alcohol at light levels; AUD + smoke = people with alcohol use disorder who smoke cigarettes; AUD + no smoke = people with alcohol use disorder who do not smoke cigarettes; abstained = people who did not drink alcohol; recurrence = people who had a recurrence of alcohol use. ^*^ denotes that the term “relapsers” or “alcoholics” from original texts was replaced. ^**^ denotes that values are presented as median (first quartile, third quartile).

### National Institute on Alcohol Abuse and Alcoholism recovery definitions

Table 3 summarizes whether studies utilized NIAAA recovery definitions based on duration of remission from AUD symptoms (i.e. no symptoms except craving) and duration of heavy drinking cessation (i.e. drinking ≤14 drinks per week or ≤4 drinks per day for men, and ≤7 drinks per week or ≤3 drinks per day for women). None of the 20 studies reported duration of remission from AUD symptoms. While all studies assessed AUD symptoms at baseline, no studies conducted diagnostic interviews at follow-up timepoints to assess whether AUD symptoms persisted; thus, duration of remission could not be determined.

**Table 3:** NIAAA recovery definitions.

Article	Duration of remission: Does not meet any DSM criteria (or equivalent) except craving	Duration of heavy drinking cessation: Men: ≤14 drinks/week or ≤ 4 drinks/day Women: ≤ 7 drinks/week or ≤ 3 drinks/day
Maillard et al. (2022)	No; met for DSM-V severe AUD at baseline	Yes; participants were considered to drink at “low-risk” levels when they consumed ≤14 drinks/week (men) or ≤ 7 drinks/week (women)
Alhassoon et al. (2012)	No; met for DSM-IV alcohol dependence at baseline	No; compared participants who abstained from alcohol to participants who resumed drinking at pre-baseline levels for ≥9 months between scans^*^
Bach et al. (2020)	No; met for DSM-IV alcohol dependence criteria at baseline	No; scans were completed during abstinence
Cardenas et al. (2007)	No; met for DSM-IV alcohol dependence at baseline	No; participants were defined as having returned to alcohol use^*^ if they drank 1–15 days prior to the follow-up scan
Demirakca et al. (2011)	No; met for DSM-IV and ICD-10 alcohol dependence at baseline	No; compared participants who abstained from alcohol to participants with alcohol dependence^*^
Durazzo et al. (2015)	No; met for DSM-IV alcohol dependence at baseline	No; scans were completed during abstinence
Durazzo et al. (2017)	No; met for DSM-IV alcohol dependence criteria at baseline	No; compared participants who abstained from alcohol to participants who returned to alcohol use^*^
Durazzo and Meyerhoff (2020)	No; met for DSM-IV alcohol dependence criteria at baseline	No; compared participants who abstained from alcohol to participants who returned to alcohol use^*^
Gazdzinski et al. (2005)	No; met for DSM-IV alcohol dependence at baseline	No; compared participants who abstained from alcohol to participants who had ≥1 drink
Gazdzinski et al. (2008)	No; met for DSM-IV alcohol dependence criteria at baseline	No; scans were completed during abstinence
Gazdzinski et al. (2010)	No; met for DSM-IV alcohol dependence at baseline	No; scans were completed during abstinence
Hoefer et al. (2014)	No; met for DSM-IV alcohol dependence criteria at baseline	No; scans were completed during abstinence
Kühn et al. (2014)	No; met for DSM-IV alcohol dependence criteria at baseline	No; scans were completed during abstinence
Mon et al. (2013)	No; met for DSM-IV alcohol dependence criteria at baseline	No; scans were completed during abstinence
Pfefferbaum et al. (1995)	No; met Research Diagnostic Criteria for alcoholism at baseline	No; compared participants who drank ≤1 drink to participants who drank ≥2 drinks before timepoint 3^*^
Pfefferbaum et al. (2014)	No; met for DSM-IV alcohol dependence at baseline	No; compared participants who abstained to participants who drank ~2.1 kg per year and participants who drank >5 kg per year
Shear et al. (1994)	No; met for DSM-III alcohol dependence at baseline	No; compared participants who abstained from alcohol to participants who drank any alcohol
van Eijk et al. (2013)	No; met for DSM-IV and ICD-10 alcohol dependence at baseline	No; scans were completed during abstinence
Wang et al. (2016a)	No; met for DSM-IV and ICD-10 alcohol dependence at baseline	No; scans were completed during abstinence
Zou et al. (2018)	No; met for DSM-IV alcohol dependence at baseline	No; scans were completed during abstinence

*Note*. ^*^denotes that the term “relapsers” or “alcoholics” from original texts was replaced.

Maillard et al. (2022) was the only study that reported duration of heavy drinking cessation using the NIAAA alcohol quantity guidelines. Indeed, all studies in the original review used recovery definitions that were strictly based on abstinence. For example, some studies required participants to maintain abstinence to remain in the study (Gazdzinski et al. 2008, Mon et al. 2012, van Eijk et al. 2013, Hoefer et al. 2014, Durazzo et al. 2015, Wang et al. 2016a, Zou et al. 2018, Bach et al. 2020). Other studies that did not require participants to remain abstinent utilized analyses that compared individuals who remained abstinent (i.e. drank zero alcoholic drinks) to individuals who had returned to alcohol use^*^ by drinking one or two drinks (Shear et al. 1994, Durazzo et al. 2017, Durazzo and Meyerhoff 2020) or consuming alcohol recently during the 1–15 days prior to the follow-up (Cardenas et al. 2007). Maillard et al. (2022) was the only study that compared drinking behaviors between individuals who drank at “low-risk” levels and individuals who returned to alcohol use^*^. Specifically, the authors defined a return to alcohol use^*^ as drinking more alcohol than the amount specified by NIAAA, a reported loss of control in alcohol consumption and inability to accurately quantify alcohol consumption, or meeting the NIAAA criteria for binge drinking. Notably, the other studies did not use the NIAAA alcohol quantity guidelines as a determination of heavy drinking nor report cessation from heavy drinking.

## Discussion

This systematic review examined whether neuroimaging studies used the NIAAA definition of recovery, which allows for a harm reduction approach to conceptualizing AUD recovery that includes people who may continue drinking alcohol (NIAAA n.d., Hagman et al. 2022). The aims of the present review were to: (i) update the search results of the original Parvaz et al. (2022) review, (ii) determine whether more recently published studies and studies from the original review utilized the NIAAA definition of recovery from AUD (i.e. duration of remission and cessation of heavy drinking), and (iii) report demographic information from new and original studies. The newly identified study by Maillard et al. (2022) aligned with previous findings in showing that participants with AUD had decreased gray and white matter volume compared to controls, and used part of the NIAAA definition of recovery (NIAAA n.d.). Participant demographic information reported in the studies was largely limited to age, sex assigned at birth, and educational attainment. Information on participant race, ethnicity, and any other sociodemographic information, such as socioeconomic status, was scarce.

Nearly all studies except Maillard et al. (2022) characterized recovery using abstinence-based definitions rather than the NIAAA definition of recovery. The inclusion criteria for some studies required participants to remain completely abstinent from alcohol (Gazdzinski et al. 2008, Mon et al. 2012, van Eijk et al. 2013, Hoefer et al. 2014, Durazzo et al. 2015, Wang et al. 2016a, Zou et al. 2018, Bach et al. 2020). A potential reason for this is that a number of studies recruited from treatment facilities (Gazdzinski et al. 2008, Mon et al. 2013, van Eijk et al. 2013, Wang et al. 2016a, Bach et al. 2020) where participants were supervised and did not have access to alcohol. However, findings from studies that recruit from treatment centers may not be generalizable to other adults who drink alcohol. Other studies separated participants into binary categories based on their alcohol use patterns, such as labeling those who consumed 1–2 alcoholic drinks as having returned to drinking^*^ (Shear et al. 1994, Durazzo et al. 2017, Durazzo and Meyerhoff 2020) and comparing those participants to others who consumed zero alcoholic drinks. However, reporting alcohol outcomes only as binary categories (any alcohol use versus no alcohol use) is limiting, as this practice obscures rich data about severity and quantity of alcohol use. Instead, measuring alcohol use as a continuous outcome would allow for research that can draw stronger associations between neuroimaging findings at different levels of alcohol use. Further, labeling participants who consumed any amount of alcohol as “relapsers” may be stigmatizing and inaccurate (Miller 2015, Hartwell et al. 2022), as using this label presumes that participants who drink any amount of alcohol are no longer in recovery if they do not actively abstain from alcohol (Witkiewitz and Tucker 2020). Lastly, no studies reported duration of remission from AUD symptoms (based on DSM-5 criteria) in accordance with the NIAAA definition of recovery at post-baseline timepoints. While all studies included a diagnostic assessment of alcohol-related symptoms at baseline, this information was not reported at later timepoints. Only neuroimaging outcomes and alcohol consumption at follow-up were reported. Thus, it could not be determined whether participants indeed remained in remission from AUD as part of their recovery.

AUD can negatively impact multiple areas of one’s life, such as impaired functioning in social, occupational, and health domains, and the severity of these consequences may vary as a function of demographic variables (Delker et al. 2016, White 2020). While these adverse consequences may include structural changes to the prefrontal cortex (Yang et al. 2016, Wang et al. 2016b, Durazzo and Meyerhoff 2020), the consequences of AUD also extend far beyond such structural brain changes. Merely quantifying the amount of gray or white matter in the brain or the amount of alcohol consumed without examining functioning in other areas of life (as defined by DSM-5 criteria) overlooks important information about how adults in recovery from AUD may exhibit improved emotion regulation or decreased impulsive behaviors (Jakubczyk et al. 2018), which may in turn be linked to improved treatment outcomes (Loree et al. 2015). Thus, reporting the decrease of AUD symptoms at subsequent timepoints throughout recovery would allow researchers to map structural brain recovery onto individuals’ real-world progress across various life domains. The NIAAA definition of recovery recognizes the importance of symptom alleviation on improving multiple life domains: “recovery is often marked by the fulfillment of basic needs, enhancements in social support and spirituality, and improvements in physical and mental health, quality of life, and other dimensions of well-being” (Hagman et al. 2022, p. 808; NIAAA n.d.). Assessing AUD symptoms longitudinally has clinical importance as this information could be helpful to those in recovery who struggle to see the potential benefits of remaining in recovery. Moreover, identifying associations between increased gray and white matter with decreases in self-reported AUD symptoms could provide important clinical guidelines for working with adults at different stages of recovery. In addition, distinguishing between demographic variables such as biological sex and gender may provide insights into structural brain recovery. There is substantial evidence that biological sex and gender can have independent and interactive effects on brain structure, function, and health outcomes (Rauch and Eliot 2022, Luckhoff et al. 2024). For example, alcohol consumption and related consequences differ between men and women (White 2020). Such differences may, in part, be attributable to gender-associated experiences and behaviors, which could also impact brain structure and neuroimaging findings (Rauch and Eliot 2022). Furthermore, disaggregating sex and gender may result in distinct neuroimaging findings (Luckhoff et al. 2024), which suggests the importance of examining both sex and gender for more nuanced interpretation of findings. Thus, our suggestion that neuroimaging studies report both sex and gender reflects an evidence-based approach aimed at improving rigor, reproducibility, and interpretability. Conflating these variables may obscure meaningful sources of variability and limit the generalizability of findings. Lastly, defining recovery only in terms of any alcohol use versus no alcohol use may perpetuate stigma and function as a treatment barrier for individuals who subscribe to a “controlled drinking” or harm reduction approach to alcohol use. Broadening the definition of recovery will increase the numbers of participants with AUD who are eligible to participate in neuroimaging research, though increased diversity in alcohol use patterns may potentially correspond to increased variation in neuroimaging findings as well. Thus, an approach to AUD recovery research that not only measures structural brain recovery, but also carefully considers how differing quantities of heavy drinking, alcohol-related symptoms, and demographic factors relate to neuroimaging findings, may provide richer data that is more inclusive of people in various stages of recovery.

While the results of this review provide important insights into existing research, there were several limitations that must be considered. First, our review was limited to articles that used structural MRI methods. Methodological concerns have been raised regarding whether structural MRI, resting-state functional MRI, and diffusion tensor imaging methods reflect true structural changes or the effects of confounding factors (Weinberger and Radulescu 2016, 2021). Furthermore, MRI is not a direct measure of brain structure or true volume (Weinberger and Radulescu 2016). Thus, an important future direction is to extend the scope of this review by assessing whether articles that use fMRI methods report NIAAA definitions of recovery and demographic information. Second, since the current review was an update of the original Parvaz et al. (2022) review, our search period was limited from May 2021 to June 2025. This short timeframe likely contributed to the limited articles that were identified in our search; conducting this search over a larger timeframe could yield a larger number of articles. Additionally, if this search were to be repeated at a future date, it is possible that the NIAAA definitions of recovery would be utilized in more articles. Third, there was variation in whether studies in the Parvaz et al. (2022) review reported effect sizes or adjusted for multiple comparisons in the neuroimaging findings. The lack of effect sizes reporting hinders the ability to appreciate the magnitude of effects, which in turn limits the ability to gage the clinical significance of study results. Further, running numerous analyses, as is standard in neuroimaging studies, without statistical adjustments may result in Type 1, or false positive, errors. Thus, future neuroimaging research in this area should aim to more consistently report effect sizes and correct for multiple comparisons, and future systematic reviews should take these statistical considerations into account when assessing the quality of studies. Lastly, most studies did not utilize NIAAA definitions of recovery (i.e. duration of remission from AUD symptoms and cessation from heavy drinking) at subsequent timepoints. Future neuroimaging research should aim to recruit individuals in various stages of recovery and record more comprehensive outcomes beyond abstinence from alcohol to improve clinical guidelines for adults at different stages of recovery.

Recovery from AUD is a dynamic and unique process marked by many changes in one’s functioning. The original Parvaz et al. (2022) review provides hope that structural recovery in the brains of those with AUD can occur relatively early in the recovery process. However, due to the nature of the included studies, this finding might be specific to individuals who are primarily abstinent from alcohol use. It remains unclear whether these findings generalize to participants who are continuing to drink alcohol, as well as participants from racially diverse and gender diverse backgrounds. Thus, more inclusive recruitment, more comprehensive reporting of demographic information, and updated research using contemporary definitions of AUD recovery are necessary. These practices may, in turn, provide stronger, evidence-based treatment recommendations for clinicians working with adults who are in recovery from AUD.

## Supplementary Material

PRISMA_2020_checklist_clean_5_agag044

## Data Availability

This systematic review does not include any original data. All data analyzed are from previously published studies, which are publicly available in the cited sources.
